# Cerebrospinal fluid-contacting nucleus mediates nociception via release of fractalkine

**DOI:** 10.1590/1414-431X20176275

**Published:** 2017-08-07

**Authors:** Q.Q. Zhou, S.S. Chen, Q.Q. Zhang, P.F. Liu, H.Z. Fang, Y. Yang, L.C. Zhang

**Affiliations:** 1 Jiangsu Province Key Laboratory of Anesthesiology, Xuzhou Medical University, Xuzhou, Jiangsu Province, China; 2 Jiangsu Province Key Laboratory of Anesthesia and Analgesia Application Technology, Xuzhou Medical University, Xuzhou, Jiangsu Province, China

**Keywords:** Cerebrospinal fluid-contacting nucleus, FKN, microglia, Neuropathic pain, Chronic constriction injury

## Abstract

Increasing evidence suggests that the cerebrospinal ﬂuid-contacting nucleus (CSF-contacting nucleus) mediates the transduction and regulation of pain signals. However, the precise molecular mechanisms remain unclear. Studies show that release of fractalkine (FKN) from neurons plays a critical role in nerve injury-related pain. We tested the hypothesis that release of FKN from the CSF-contacting nucleus regulates neuropathic pain, in a chronic constriction injury rat model. The results show that FKN is expressed by neurons, via expression of its only receptor CX3CR1 in the microglia. The levels of soluble FKN (sFKN) were markedly upregulated along with the increase in FKN mRNA level in rats subjected to chronic constriction injury. In addition, injection of FKN-neutralizing antibody into the lateral ventricle alleviated neuropathic pain-related behavior followed by reduction in microglial activation in the CSF-contacting nucleus. The results indicate that inhibition of FKN release by the CSF-contacting nucleus may ameliorate neuropathic pain clinically.

## Introduction

The CSF-contacting nucleus is a special nucleus found in the brain, which was initially discovered and named by our group worldwide. This nucleus is composed of special neurons called the distal cerebrospinal ﬂuid-contacting neurons whose bodies are in the parenchyma and processes extend into the CSF in the cavity. They are primarily located in the ventral periaqueductal central gray (PAG) area of the medulla and brainstem (1). Recently, our group showed that direct injection of cholera toxin subunit B into the lateral ventricle (LV) is a reliable and specific method of labeling the CSF-contacting nucleus (2). The unique anatomical structure of the CSF-contacting nucleus indicates that it is the only area within the CNS involved in bidirectional signal transduction and transport of bioactive substances between the brain parenchyma and CSF (3–5). Furthermore, we previously reported that the CSF-contacting nucleus may participate in the signal transduction and regulation of neuropathic and inﬂammatory pain (6–8). Nevertheless, the precise molecular mechanisms remain indistinct.

Neuropathic pain refers to pain arising as a direct consequence of a lesion or disease affecting the somatosensory system. Current treatments of neuropathic pain are inadequate due to insufficient understanding of the mechanisms. Recent findings suggest that glial cells play a vital role in the processes underlying neuropathic pain. Peripheral nerve injury triggers activation of microglia and astrocytes in the spinal cord. Subsequently, these cells release a series of pro-nociceptive mediators to regulate the activity of neurons (9,10). The CX3CL1 is a chemokine released from neurons during the neuronal-to-microglial signaling (11). As the only member of CX3C family, CX3CL1 (Fractalkine, FKN) contains two cysteines separated by three amino acids (12), and it exhibits unique traits. First, FKN binds only to one known receptor CX3CR1. In addition, FKN exists in two different forms: a membrane-bound protein mediating integrin-independent adhesion and a soluble form containing a chemokine domain that have chemo-attractive activity. Furthermore, FKN is the only extracellular chemokine in neurons, and the soluble FKN (sFKN) activates nearby glia (13,14). Many published articles have demonstrated that FKN/CX3CR1 is a key signaling pair during neuropathic pain states (14–21).

The present study was designed to detect the distribution of FKN and CX3CR1 within the CSF-contacting nucleus in normal rats, and their expression in neuropathic pain rat model. We also evaluated the effect of FKN-neutralizing antibody on neuropathic pain-related behavior and microglial activation. This study provides insight into the physiology of neuropathic pain in the brain, by analyzing the expression of FKN in the CSF-contacting nucleus.

## Material and Methods

### Animals

Adult male Sprague-Dawley rats, weighing 250-300 g, were purchased from the Experimental Animal Center of Xuzhou Medical University. Animals were caged under controlled laboratory conditions (23±1°C) and exposed to 12/12 h dark/light cycle and food and water *ad libitum*. At least 1 week was allowed to let the animals acclimatize to the housing environment. The procedures were approved by the Committee for the Ethical Use of Laboratory Animals, Xuzhou Medical University (SYXK 2015-0030). The study was compliant with the guidelines of the International Association for the Study of Pain.

### Surgical procedures

Brieﬂy, rats were treated with 10% chloral hydrate (300 mg/kg, *ip*) to induce anesthesia. Blunt dissection was used to expose the left sciatic nerve at the mid-thigh level. Using 4-0 braided silk thread (Ethicon Inc., Belgium), four ligatures were created loosely around the nerve with approximately 1 mm spacing. The incision was sealed in layers. In sham-operated rats, identical procedures were performed except sciatic nerve ligations, and in naïve rats, no processing was performed. Animals were kept warm and allowed to recover from anesthesia. When the rats were completely awake, they were returned to the cage to rest.

### Behavioral tests

The animals were acclimatized to the environment for 30 min before behavioral testing, which was conducted blindly.

Thermal pain was evaluated using the IITC Plantar Analgesia Meter (IITC Life Science Inc., USA). Thermal withdrawal latency (TWL) was measured in terms of delayed reﬂex from the start of radiant heat exposure until hind paw withdrawal. The strength of the heat stimulus was controlled to yield a baseline thermal withdrawal latency of about 15 s in normal and sham-operated rats. Exposure was limited to 20 s, to avoid tissue damage.

Mechanical allodynia was assessed using von Frey filaments (Semmes-Weinstein Monofilaments; North Coast Medical, USA). The paw withdrawal threshold (PWT) was evaluated by steadily controlling the stimulus strength (the "up-and-down" method).

### Intracerebroventricular injection

Rats were exposed to 10% chloral hydrate (300 mg/kg, *ip*) to induce anesthesia followed by immobilization in a digital stereotaxic instrument (Stoelting, USA). A hole was drilled in the skull after a midline incision. Drugs were injected slowly with a Hamilton microsyringe into one of the LV under stereotactic guidance (Brega: 1.2±0.4 mm, deep: 3.2±0.4 mm, right to median sagittal plane: 1.4±0.2 mm) over 15 min. An additional 15 min was allowed to prevent drug diffusion. Animals were kept warm and allowed to recover from anesthesia. When the rats were completely awake, they were returned to the cage to rest.

### Drugs and treatments

Cholera toxin subunit B (CB) was obtained from Absin Bioscience Inc. (China). Anti-rat FKN-neutralizing antibody (AF537) and normal goat IgG were acquired from R&D Systems. We administered 900 ng/3 μL CB into rat LVs to label the CSF-contacting nucleus. An amount of 10 μg/μL of rat FKN-neutralizing antibody or normal goat IgG were administered into the LV on day 14 after CCI procedure.

### Immunoﬂuorescence

Immunoﬂuorescence procedures were performed as described previously. Brieﬂy, the expression of FKN was analyzed by incubating sections of the CSF-contacting nucleus (40 μm) with goat anti-CB (1:400; Sigma, USA) and rabbit anti-CX3CL1 (1:500; Abcam, UK). The CX3CR1 expression was assessed by incubating sections of the CSF-contacting nucleus (40 μm) with goat anti-Iba-1 (1:200; Abcam) and rabbit anti-CX3CR1 (1:200; Abcam). Microglial expression was evaluated by incubating sections of the CSF-contacting nucleus (40 μm) with rabbit anti-CB (1:1000; Abcam) and goat anti-Iba-1(1:200; Abcam). All the specimens were incubated for 24 h at 4°C, followed by reaction with donkey anti-goat IgG conjugated with Alexa 546 (1:200; Life Technologies, USA) and donkey anti-rabbit IgG conjugated with Alexa 488 (1:200; Life Technologies) in PBST for 1 h at 37°C. The sections were washed, transferred onto a slide and cover-slipped. Images were visualized under a confocal laser microscope (FV1000; Olympus, Japan).

### Real-time PCR

The CSF-contacting nucleus was removed on days 3, 7, 14, and 21 after CCI surgery. The samples were treated with 500 μL of TRIzol (Invitrogen, China). The total RNA was extracted using routine procedures. Real-time PCR was conducted using the following primers specific for rat FKN and GAPDH: FKN, forward: 5-GCCACAAGATGACCTCGCCAAT-3, reverse: 5-TGCTGTCTCGTCTCCAGGATGA-3; GAPDH, forward: 5-GGCCTTCCGTGTTCCTACC-3, reverse: 5-CGCCTGCTTCACCACCTTC-3.

### Western blot

After the behavioral tests, the CSF-contacting nucleus was isolated and stored at -80°C. Samples were treated with a lysis buffer comprising protease inhibitors and phosphatase inhibitors. The protein concentrations of the lysate were analyzed using a BCA Protein Assay, followed by SDS/PAGE. The separated proteins were transferred to nitrocellulose membranes and incubated with primary antibodies against FKN (1:1000; Abcam) and GAPDH (1:3000; Bioworld, China). After incubation for 1 h at room temperature with secondary anti-rabbit IgG antibody (1:1000, Sigma), the grayscale images of the western blot were analyzed using ImageJ software (NIH, USA).

### Quantitative assessment

Quantitative assessment of microglia within the CSF-contacting nucleus was conducted by counting the positive test results within a segment of the nucleus. A box measuring 10^4^ μm^2^ was placed on the areas to the left and right CSF-contacting nucleus. The number of cells testing positive for Iba-1 within this area was determined. This measurement protocol was carried out on three specimens in each animal.

### Statistical analyses

Results are reported as means±SE. Real-time PCR and western blot data were analyzed using one-way ANOVA followed by least significance difference (LSD) test. Two-way ANOVA combined with LSD was used to compare the activation of microglia after drug injection. Behavioral results after CCI and drug treatment were analyzed by ANOVA with repeated measurements test. A value of P<0.05 was considered to be statistically significant.

## Results

### Thermal hyperalgesia and mechanical allodynia

**Figure 1.**
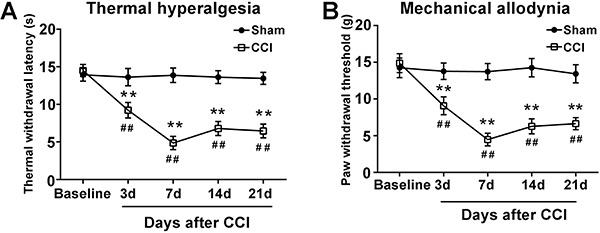
Behavioral testing of chronic constriction injury (CCI) rats. *A*, Duration of thermal withdrawal latency (F=61.487); *B*, paw withdrawal threshold (F=53.652). **P<0.01 compared with sham group at the same time point; ^##^P<0.01 compared with baseline, n=7 per group (ANOVA with repeated measurements).

The TWL of the CCI group significantly decreased from day 3 to day 21 after surgery compared with the baseline and sham group, with the minimum value recorded on day 7 (Figure 1A). Similar results were obtained with the PWT testing (Figure 1B). Thermal hyperalgesia and mechanical allodynia were initiated on day 3 after CCI until day 21, peaking on day 7. However, no obvious hyperalgesia and allodynia were observed in the sham-operated rats.

### Distribution of FKN and CX3CR1

**Figure 2.**
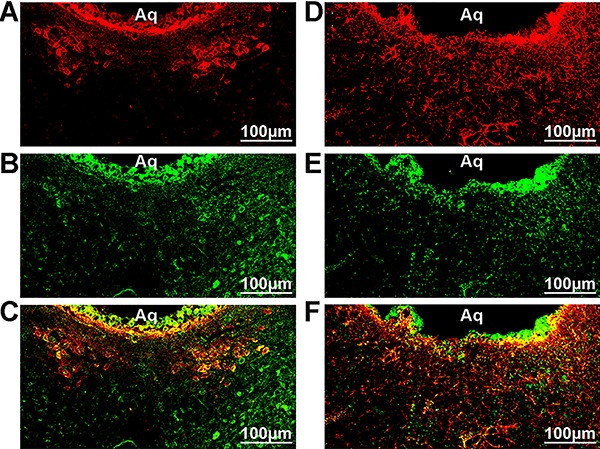
Expression of fractalkine (FKN) and CX3CR1 in the CSF-contacting nucleus. *A*-*C*, Cholera toxin subunit B-labeled positive neurons (red) and FKN-immunoreactive neurons (green) were co-expressed in specific neurons (yellow in merged image). *D*-*F*, Colocalization of Iba-1 (red) and CX3CR1 (green) immunoreactivity (yellow in merged image). n=3 per group, Scale bar =100 μm. Aq: aqueduct. CSF: cerebrospinal fluid.

CB-labeled positive neurons in red were distributed uniformly in the ventral PAG. Dual labeling of neurons (yellow) demonstrated that FKN-immunoreactive neurons (green) were frequently colocalized with CB-labeled cells (Figure 2A-C), which was consistent with previous reports (20,22) suggesting that FKN was expressed by neurons. In addition, we used Iba-1 as the microglial marker, and confirmed the expression of CX3CR1 (green) in microglia (red) within the CSF-contacting nucleus (Figure 2D-F), which has been extensively reported (17,18).

### FKN mRNA expression in the CSF-contacting nucleus under neuropathic conditions

**Figure 3.**
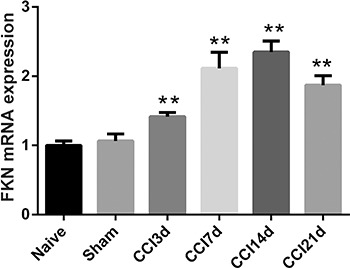
Fractalkine (FKN) mRNA expression was increased after chronic constriction injury (CCI) surgery. Real-time PCR was conducted with total RNA samples extracted from the CSF-contacting nucleus in naive, sham, and CCI rats on days 3, 7, 14, and 21. **P<0.01 *vs* sham group, n=5, F=48.2 (one-way ANOVA, LSD test). CSF: cerebrospinal fluid.

A marked up-regulation of FKN mRNA expression was observed in CCI group within the CSF-contacting nucleus, starting from day 3, peaking on day 14, and persisting until day 21 after CCI surgery. However, in the sham group, there was no significant variation (Figure 3).

### Up-regulation of FKN release in the CSF-contacting nucleus under neuropathic conditions

**Figure 4.**
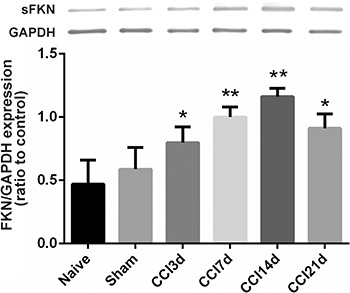
Western blot of soluble fractalkine (sFKN) expression. Protein samples were isolated from the CSF-contacting nucleus in naive, sham, and chronic constriction injury (CCI) rats on days 3, 7, 14, and 21. *P<0.05 *vs* sham group, **P<0.01 *vs* sham group, n=5, F=19.065 (one-way ANOVA, LSD test). CSF: cerebrospinal fluid.

Western blot revealed a specific band (about 50 kDa) in the CSF-contacting nucleus using a FKN antibody. The intensity of the FKN band significantly increased after CCI surgery starting on day 3, peaking on day 14, and sustained until day 21 after CCI. Furthermore, no significant change was observed in sham group (Figure 4).

### Effect of FKN-neutralizing antibody on pain

**Figure 5.**
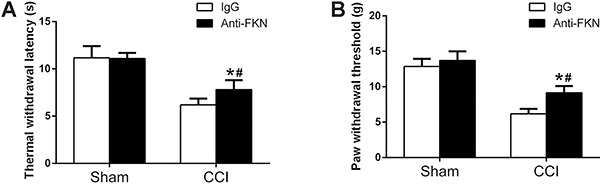
Fractalkine (FKN)-neutralizing antibody prevented chronic constriction injury (CCI)-induced pain. *A*, Thermal hyperalgesia after administration (F=58.575). *B*, Paw withdrawal threshold (F=92.935). *P<0.05 *vs* CCI + IgG group, ^#^P<0.05 *vs* Sham + Anti-FKN group, n=8 per group (two-way ANOVA, LSD test).

The sFKN expression within the CSF-contacting nucleus peaked on day 14. Therefore, a single dose of FKN-neutralizing antibody (10 μg, partial inhibition) into LV was administered at this time (day 14) to pharmacologically verify our hypothesis that release of FKN from the CSF-contacting nucleus is involved in neuropathic pain, especially the maintenance of neuropathic pain. TWL and PWT were determined 24 h after administration. The results showed that FKN-neutralizing antibody significantly lowered CCI-induced thermal hyperalgesia and mechanical allodynia after treatment, while it had no effect on sham-operated rats (Figure 5).

### Enhanced microglial response was attenuated by FKN-neutralizing antibody

**Figure 6.**
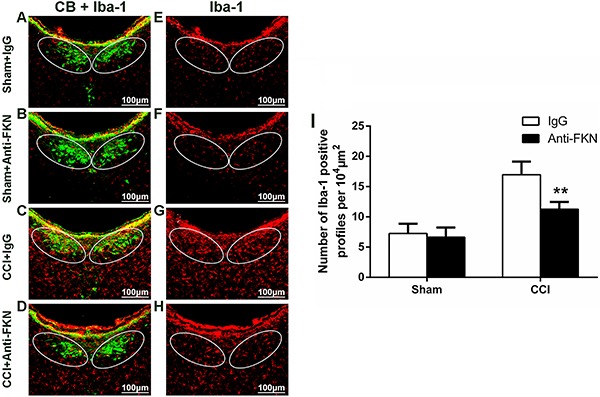
Fractalkine (FKN)-neutralizing antibody attenuated the microglial response following chronic constriction injury (CCI). CB-labeled neurons (green in *A*-*D*) were used to locate the CSF-contacting nucleus. The cells testing positive for Iba-1 (red in *E*-*H*) within the box in the areas to the left and the right of CSF-contacting nucleus were counted. *I*, Quantification of Iba-1 immunoreactivity. **P<0.01 *vs* the CCI + IgG group, n=5 per group, F=240.048 (two-way ANOVA, LSD test). Scale bar =100 μm. CSF: cerebrospinal fluid.

Rats injected with FKN-neutralizing antibody showed significantly reduced number of microglia positive for Iba-1 compared with IgG-treated animals in the CCI group, while it had no effect on sham-operated rats (Figure 6).

## Discussion

Research has shown that the CSF-contacting nucleus (1) plays a critical role in the transmission and regulation of pain. The brainstem is a critical component of the descending modulatory systems. The CSF-contacting nucleus is present in the ventral PAG of the brainstem (1). It is a key player in the downstream pain regulation system (3,23). Considering that CB-HRP can specifically label the CSF-contacting nucleus, our group has successfully ablated the CSF-contacting nucleus 7 days after intraventricular injection of cholera toxin subunit B-saporin. The ablation resulted in hypersensitivity to acute nociceptive stimulation, exacerbated behavioral changes to chronic immobilization, and enhanced sodium absorption (3–5). We also showed that variations in several substances within the CSF-contacting nucleus, such as substance P, ASIC3, mTOR, ERK1/2 and Wnt5a regulate pain (6–8). Our current study provides strong evidence that FKN expressed in the CSF-contacting nucleus is released during neuropathic pain. Furthermore, injection of FKN-neutralizing antibody into LV resulted in amelioration of neuropathic pain.

Pain is associated with signal transduction in the immune system. Signaling across immunocompetent cells is a critical component in the pathophysiology of chronic pain. In addition to neurotransmitters released by the neurons, immune mediators released from other cells such as microglia and astrocytes also enhance neuronal excitability across the nociceptive system (24). During peripheral nerve injury, activated glial cells release TNF-α and IL1-β, which affect the neurons and amplify the pain response. FKN, the only member of CX3C chemokine family, play a critical role in the spread of nociceptive signaling from neuron to glia (14).

Previous studies strongly suggested that FKN was expressed by neurons and not endothelium, in the spinal cord (20) and the brain (22). However, its receptor CX3CR1 is expressed by microglia (17). Our results support FKN expression by the CSF-contacting nucleus, and the expression of its receptor CX3CR1 in the microglia. The release of membrane-bound FKN into soluble forms is a key mechanism regulating FKN signaling. CatS (cathepsin S) is a lysosomal cysteine protease that mediates the release of sFKN (∼50 kDa) during chronic pain (19,20). Following nerve injury, the sFKN levels in CSF are significantly increased (20). To our knowledge, studies investigating FKN expression in brain during pain are limited. Western blot revealed a specific band (about 50 kDa) of sFKN. The expression levels of sFKN were markedly up-regulated along with the increase in FKN mRNA level within the CSF-contacting nucleus of CCI rats.

Intrathecal administration of the sFKN (14–17), but not full-length FKN (14) induces thermal hyperalgesia and mechanical allodynia, which is blocked by pretreatment with neutralizing antibody against CX3CR1 (15,16) or neutralized in CX3CR1 knockout mice (18). Studies have demonstrated that sFKN activates CX3CR1 and phosphorylation of p38 mitogen-activated protein kinase (MAPK) in microglia (17,19), which triggers the synthesis of TNF-α, IL-1β, and IL-6 (16,21) that amplify nociception. Interference of spinal FKN/CX3CR1 signaling is a potential therapeutic target in chronic pain. Treatment with FKN- or CX3CR1-neutralizing antibodies ameliorates neuropathic and inﬂammatory pain (15,17,21). Considering the unique anatomical features of the CSF-contacting nucleus, we believe that drugs injected into the LV are therapeutically effective. Therefore, we injected FKN-neutralizing antibody into the LV on day 14. Alleviation of neuropathic pain-related behavior by the FKN-neutralizing antibody was accompanied by a reduction in microglial activation in the CSF-contacting nucleus.

In conclusion, this study suggests that FKN within the CSF-contacting nucleus was a crucial element linking peripheral nerve injury to microglial activation. The CSF-contacting nucleus regulates neuropathic pain by liberating sFKN. We believe that the inhibition of FKN release is a new approach to treat neuropathic pain.
